# Knowledge regarding Basic Life Support among Health Care Workers of the Hospital of Nepal

**DOI:** 10.1155/2023/9936114

**Published:** 2023-01-05

**Authors:** Gautam Prasad Chaudhary, Kalyani Sah, Juhi Malla, Nikita Das, Sabina Chaudhary, Indrajeet Chaudhary, Jitendra Pandey

**Affiliations:** ^1^Department of Pharmacy, Crimson College of Technology, Pokhara University, Devinagar-11, Butwal 32900, Nepal; ^2^Department of Medical-Surgical Nursing, Unique College of Medical Science and Hospital Pvt. Ltd., Purbanchal University, Saptari 56400, Nepal

## Abstract

Basic life support refers to a sequence of care provided to patients who are experiencing respiratory arrest, cardiac arrest, or airway obstruction. It is a specific level of prehospital medical care provided by the trained responders, including emergency medical technicians, in the absence of advanced medical care to maintain the patient's life. BLS course trains participants to promptly recognize several life-threatening emergencies, give high-quality chest compressions, deliver appropriate ventilations, and provide early use of an AED. According to American Heart Association (AHA) guidelines, every missed minute in applying defibrillation in a cardiac arrest decreases the survival rate by 70%–10%. According to European Resuscitation Council (ERC), early resuscitation and prompt defibrillation (within 1-2 minutes) can result in >60% survival. A quantitative, descriptive study design is used in this study. A purposive sampling technique was used, and the sample size was 95. A self-structuredclose-ended questionnaire was used to assess the level of knowledge of the participants. The finding revealed that among 95 participants, only 12% had adequate, 55% had moderate, and 32% had inadequate knowledge about Basic Life Support. The study showed that knowledge among healthcare workers about basic life support is insufficient for the majority of participants. There is a significant association between dependent and independent variables.

## 1. Introduction

Basic Life Support (BLS) refers to a sequence of care provided to patients who are experiencing respiratory arrest, cardiac arrest, or airway obstruction [1]. It is a specific level of prehospital medical care provided by the trained responders, including emergency medical technicians, in the absence of advanced medical care to minimize the patient's critical condition. BLS course trains participants to promptly recognize several life-threatening emergencies, give high-quality chest compressions, deliver appropriate ventilations, and provide early use of an automated external defibrillator (AED) [2]. Basic Life Support (BLS) is a lifesaving intervention in a premedical facility. Adequate knowledge and awareness about BLS and Cardiac pulmonary resuscitation (CPR) are mandatory for the healthcare worker working in a hospital [3, 4]. Cardiac arrest or cardiopulmonary arrest can leave the victims with severe morbidities or lead to death if not attended to instantly or treated promptly. Early identification and intervention of cardiac arrest victims by performing CPR are the cornerstones of BLS, which helps sustain the patient's life until definitive medical care arrives and the patient is transferred to a hospital setting for further advanced management. BLS's purpose is to maintain the airway, breathing, and circulation through CPR. CPR is an emergency procedure for the restoration of cardiac and respiratory functions after cardiac arrest [5, 6]. It consists of a combination of mouth-to-mouth ventilation and external chest compression that is given to a person thought to have been in cardiac arrest [7]. It is performed by anyone who knows how to do it anywhere and immediately, without equipment and protective devices. It may include considerations of patient transport such as the protection of the cervical spine and avoiding additional injuries through splinting and immobilization [8]. It includes psychomotor skills for performing high-quality cardiopulmonary resuscitation (CPR), using an automated external defibrillator (AED), and relieving an obstructed airway for patients of all ages. American Heart Association (AHA) guidelines suggest that every missed minute in applying defibrillation in a cardiac arrest decreases the survival rate by 7%–10% [9]. According to European Resuscitation Council (ERC), early resuscitation and prompt defibrillation (within 1-2 minutes) can result in >60% survival [10].

## 2. Materials and Methods

A descriptive study design was used on the healthcare workers of the hospital. The sample size was calculated by using the *z*^2^pq/*L*^2^ formula where *z* = tabulated value in the 95% level of significance (1.96), *p* = prevalence rate = 52%, *q* = 1 − *p* = 1–52/100 = 0.48, *L* = allowable error = 10% = 10/100 = 0.1 the sample size found to be was 96 [11–13]. The study used a technique called purposive sampling. Knowledge of basic life support was the study's dependent variable, and social-demographic factors such as age, gender, religion, place of residence, and educational attainment of healthcare workers with valid work permits were the study's independent variables. Both genders were included in the study, whereas medical officers, consultants, and hospital incharge were excluded from the study.

The self-structured closed-ended questionnaire was used to collect data which contains two parts; part I consists of socio-demographic data such as age, gender, work experience, previous training, and education level, and part II consists of items related to knowledge regarding basic life support. A scoring key was designed where each correct answer was awarded 1 mark and the wrong answer 0. Thus, the item maximum score was 30 and the minimum was 0. Consequently, the highest total knowledge score for health workers was 30. Since health workers should have sufficient knowledge in this very critical area, knowledge scores of above 23 or >75% were considered “adequate” knowledge, scores 15–23 or 50–75% were considered “moderate” knowledge, and scores of below 15 or <50% were considered as “inadequate” knowledge.

Before actual data collection, the instrument was pretested on 10% of the total sample size. Ethical consideration was taken from Purbanchal University and informed consent was designed; confidentiality of the information was maintained throughout the study. Collected data were checked and rechecked for their completeness for missing items before their processing, and that data were edited, coded, and entered into computer base software using SPSSV20 by using both descriptive and inferential statistics [14, 15].

## 3. Results

### 3.1. Distribution of Sample According to Demographic Data

The majority of respondents were of the 30–40 age group which is 52.63% followed by 33.63% of the 20–30 age group and at least 13.68% of the 40–50 age group. The mean age for the study participants was 33 of 95 respondents, where 85 (89.47%) were females and 10 (10.53%) were males. Our study reveals that 56 (58.95%) respondents were proficient in certificate level in nursing (PCL Nursing) followed by auxiliary nurse midwife (ANM) 21 (22.11%), followed by health assistant (HA) and community medicine assistant (CMA) 9 (9.47%). Based on work experience, our study demonstrates that the maximum year of experience is of 2-3 years 40 (42.11%), followed by 3-4 years' experience was 25 (26.32%), <1 year was about 17 (17.89%), and 1-2 years' experience was 13 (13.68%). The majority of participants have not received any training regarding BLS; only 15 (15.79%) respondents have received training and 80 (84.21%) have not received any training. (Table 1).

Table 2 represents that more than half of the total respondents 52 (55%) of respondents had moderate knowledge followed by inadequate knowledge with 32%, and adequate knowledge which was 12% (Table 2).

### 3.2. Association between the Level of Knowledge and Socio-Demographic Variables

Table 3 shows that there is a strong association between the age group and the level of knowledge. *P* value by the chi-square test was found to be 0.021637 ^*∗*^*P* value <0.05 considered to be statically significant (Table 3), but it is found that there is no association between gender and level of knowledge. The *P* value by the chi-square test was found to be 0.990895. *P* value >0.05 considered to be statically nonsignificant. Our study revealed that there is no association between the qualification of respondents and their level of knowledge; the *P* value by the chi-square test was found to be 0.9885 and is nonsignificant. Table 3 depicts that there is no association between the work experience of respondents and their level of knowledge as the *P* value by the chi-square test is 0.840103 and it shows that it is nonsignificant. The present study demonstrates that there is a strong relationship between the previous training of respondents and their level of knowledge as the *P* value is 0.026244 which is highly significant.

## 4. Discussion

According to studies and reports, RTA (Road Traffic Accidents), cut injuries, and sudden cardiac deaths are the major life-threatening risks in Nepal. The risk of road traffic death is 3 times higher in low-income countries (8.3 deaths per 100,000 populations) when compared to high-income countries [16]. In all of the major trauma and cardiac arrest or emergency cases, BLS support is necessary to save a life in a prehospital facility. The BLS program is designed to deliver knowledge to a wide range of healthcare professionals about several life-threatening emergencies. It also demonstrates training to provide CPR, use an AED, and relieve choking in a safe, timely, and effective manner to treat those emergencies. It is mandatory for all healthcare professionals to have comprehensive knowledge and skill about BLS [17, 18]. Hence, all healthcare institutes should emphasize training the healthcare professionals working in hospitals for the BLS.

Our study included 95 respondents; the majority of respondents were of the 30–40 age group followed by the 20–30 age group and at least the 40–50 age groups. The mean age of the study participants was found to be 33 years. These findings are comparable to a previously published similar study, which reported that most of their respondents were between 23 and 40 years old. In our study, the comparison of knowledge among the various age groups revealed statistical significance [19].

In our study, the majority of respondents were females (89.47%) (Table 1). This finding was in accordance with the results reported by another researcher from Botswana [20]. In their study on cardiopulmonary resuscitation knowledge and skills of registered nurses, they detected that most of the studied group were females. Another study conducted in India (focused on knowledge and perspective on CPR among staff nurses) declared that most of their study group was female [21]. The current work revealed no significant differences in BLS knowledge level and gender (Table 3); this was in accordance with a study from Botswana [20].

In the current study, more than half of the respondents, that is, 56 (58.95%), were proficient in the certificate level in nursing (PCL Nursing) followed by auxiliary nurse midwife (ANM), that is, 21 (22.11%), and then health assistant (HA) and community medicine assistant (CMA), that is, 9 (9.47%). Moreover, our study concluded that there was no association between the BLS knowledge level and academic qualification. It is to be noted that our results were supported by an investigation conducted by researchers from Nepal. In their study on knowledge regarding basic life support among nurses of a tertiary-level hospital, there was no association between nurses' knowledge and their academic qualification [22].

Based on work experience, our study demonstrates that the maximum year of experience is of 2-3 years 40 (42.11%), followed by 3-4 years' experience was 25 (26.32%), <1 year was about 17 (17.89%), and 1-2 years' experience was 13 (13.68%). There are no significant differences in the BLS knowledge level and years of experience. The majority of participants have not received any training regarding BLS. Only 15 (15.79%) of respondents have received training and 80 (84.21%) have not received any training. In the current study, we found a significant association between the knowledge level and previous training (Table 3). Also, our result is supported by the findings of different studies [23–25], which reported that BLS training is highly effective and made statically significant.

## 5. Conclusion

The results of this study indicate that knowledge of BLS among health professionals from nursing, health assistants, auxiliary nursing midwives, and community medicine assistant at hospitals is poor which becomes a critical issue and needs to be improved in the future. BSL training and clinical exposure influence the retention of knowledge; there is a need for all healthcare professionals to have some standard training and assessment. The research study was conducted to assess the knowledge regarding Basic Life Support and healthcare workers in hospitals. On the basis of the findings of the study, some recommendations such as a comparative study can be conducted, and a similar study can be conducted to find out the effectiveness of awareness and knowledge-sharing programs regarding Basic Life Support.

## Figures and Tables

**Table 1:** Distribution of sample according to demographic data.

	Respondents (*n*)	Respondents (%)
*Age group*		
20–30	32	33.63
30–40	50	52.63
40–50	13	13.68

*Gender*		
Male	10	10.53
Female	85	89.47

*Qualification*		
Proficiency certificate level in nursing (PCL nursing)	56	58.95
Health assistant (HA)	9	9.47
Auxiliary nurse midwife (ANM)	21	22.11
Community medicine assistant (CMA)	9	9.47

*Year of experience*		
<1 year	17	17.89
1-2 year	13	13.68
2-3 year	40	42.11
3-4 year	25	26.32

*Previous training*		
Yes	15	15.79
No	80	84.21

*n* = number, % = percentage.

**Table 2:** Distribution of participants based on the level of knowledge.

SN	Level of knowledge	Score (maximum = 30)	Respondent (*n*)	Respondent (%)
1	Adequate knowledge	Above 23 or >75%	12	12
2	Moderate knowledge	15–23 or 50–75%	52	55
3	Inadequate knowledge	Below 15 or <50%	31	32

**Table 3:** Association between demographic variables and knowledge level.

	Inadequate knowledge	Moderate knowledge	Adequate knowledge	*χ* ^2^	*P* value
*Age group*					
20–30	10	13	9	**17.91**	**0.0216 ** ^*∗*^
30–40	13	35	2		
40–50	8	4	1		

*Gender*					
Male	**3**	5	**2**	**0.552**	**0.9908 ** ^**NS**^
Female	28	47	10		

*Qualification*					
Proficiency certificate level in nursing (PCL nursing)	11	23	9	**3.150**	**0.9885 ** ^**NS**^
Health assistant (HA)	3	5	3		
Auxiliary nurse midwife (ANM)	11	9	6		
Community medicine assistant (CMA)	5	6	4		

*Year of experience*					
<1 year	11	7	3	**6.47**	**0.8401 ** ^**NS**^
1-2 year	12	6	2		
2-3 year	9	12	6		
3-4 year	8	12	7		

*Previous training*					
Yes	3	16	26	**12.71**	**0.0262 ** ^*∗*^
No	12	26	12		

(*χ*^2^ = Chi-square test; *P* value by the chi-square test. *P* value <0.05, considered to be statically significant, *P* value >0.05, the null hypothesis is accepted, NS-statically nonsignificant. Age and prior training are statistically significant predictors of respondents' knowledge level, whereas other factors such as gender, education, and years of experience are statistically insignificant.

## Data Availability

All the data used to support the result of this research are available from the corresponding author.

## Supplementary Materials

Supplementary Materials A list of the self-structuredclosed-ended questionnaires used to access the knowledge regarding basic life support among healthcare workers is presented in Supplementary questions S1.
