# Supplementary material for: Knowledge regarding Basic Life Support among Health Care Workers of the Hospital of Nepal
Source: J Healthc Eng. 2023 Jan 5;2023:9936114. doi: 10.1155/2023/9936114 (PMC9836805; doi:10.1155/2023/9936114)
Supplement: Supplementary Materials — A list of the self-structuredclosed-ended questionnaires used to access the knowledge regarding basic life support among healthcare workers is presented in Supplementary questions S1. [file 9936114.f1.docx]

Supplementary questions S1. List of the self-structured closed-ended questionnaires used to access the knowledge regarding basic life support among health care workers

1. The abbreviation of BLS is… [1].

a. Best Life Support

b. Basic Life Support

c. Basic Lung Support

d. Basic Life Services

2. The correct location for chest compression [1].

a. The left side of the chest

b. The right side of the chest

c. Centre of the chest on the lower half of the breastbone

d. Xiphisternum

3. The abbreviation AED is… [1].

a. Automated External Defibrillator

b. Automated Electrical Defibrillator

c. Advanced Electrical Defibrillator

d. Advanced External Defibrillator

4. The full form of EMS stands for… [1].

a. Effective Medical Services

b. Emergency Management Services

c. Emergency Medical Services

d. External Medical Support

5. The correct rate or speed that should use to perform compression for the adult victim during cardiac arrest is…[1].

a. at least 100 / min

b. approximately 100 / min

c. 80 / min

d. 120 / min

6……………………….is the foundation of CPR [2].

a. Teamwork

b. Rapid activation of EMS

c. Maintaining an open airway

d. Chest compressions

7. The main purpose of chest compression is.. [3].

a. provide ventilation

b. empty the lung of dead air

c. creates pulse

d. create blood from the heart

8. The correct sequence of resuscitation in BLS is…. [4].

a. Compressions, airway, breathing

b. Airway, breathing, compression

c. Breathing, airway, compressions

d. Airway, compression, breathing

9. The ratio of chest compression to breath in adults is.. [4].

a. 20:2

b. 30:2

c. 40:2

d. 25:2

10. The correct depth of chest compressions for adults is… [4].

a. 5-6 cm

b. 4-5 cm

c. 6-7 cm

d. 2-4 cm

11. ..................... should be palpated on an unconscious adult during CPR [4].

a. Carotid pulse

b. Radial pulse

c. Femoral pulse

d. Brachial pulse

12. The victim _________ needs CPR immediately [5].

a. with a pulse and who is having trouble breathing.

b. with chest pain and indigestion.

c. who is unresponsive, with no normal breathing and absent/inadequate pulse.

d. who is unresponsive but breathing inadequately.

13. The correct place to place hands while performing chest compression on an adult is …[6].

a. on the lower half of the breastbone

b. in the center of the breastbone

c. on the upper portion of the abdomen

d. on the upper half of the breastbone

14. The correct technique to open the airway in case of neck injury is… [7].

a. head tilt-chin lift

b. abdominal thrust

c. jaw thrust

d. both b and c

15. We perform head tilt and chin lift maneuvers by [8].

a. place the finger on the bony part of the lower jaw and tilt the head back.

b. place your finger on the angle of the jaw and tilt the head forward.

c. place your hand on the patient’s head and apply pressure so the head is stabilized.

d. place your hand on the patient forehead and apply pressure to the angle of the head back.

16. The total cycles that CPR consists of in two minutes are… [9].

a. 4 cycles

b. 10 cycles

c. 5 cycles

d. None of the above

17. The sentence that describes correct hand placement during CPR on a 2-year-old is.. [10].

a. Place one palm or both palms one over the other midline on the lower sternum between the nipples.

b. Encircle the torso with both hands with thumbs positioned 1 cm below the nipples on the sternum

c. Place two fingers on the sternum. One between the nipple line and the other 1 cm below

d. None of the above

18. We can use a defibrillator on a person for ______ times [11].

a. 3-4

b. 1-2

c. more than 5

d. more than 7

19. Maximum joules used while defibrillation is….[12].

a. 60-100 joules

b. 120-200 joules

c. 220-280 joules

d. 300-360 joules

20. The rescuer should place their hand on ______ while performing the abdominal thrust while choking [13].

a. Right below the heart

b. Right above the heart

c. Right below the belly button

d. Right above the belly button

21. The sign of airway obstruction is…. [14].

a. Poor air exchange

b. high-pitched noise while inhaling

c. inability to speak

d. all of the above

22. The minimum age to use Adult AED pads is…. [15].

a. 8 years old

b. 12 years old

c. 18 years old

d. 21 years old

23. The ideal position for you around the patient during providing CPR is…. [16].

a. Above the patient's head

b. At the patient's feet

c. At the patient's side

d. Straddling the patient

24. As you perform 2-person CPR on a patient in cardiac arrest, each breath should be delivered over……………. seconds [17].

a. 1

b. 2

c. 3

d. 5

25. While performing two-rescuer CPR, __________ we should switch roles [18].

a. After every cycle of CPR

b. After every two cycles of CPR

c. After every five cycles of CPR

d. After every ten cycles of CPR

26. You just performed 5 cycles of CPR on an adult. You reassess for a pulse. No pulse is present. Your next step is [19].

a. Search for an AED

b. call 911

c. immediately reinitiates CPR

d. none of the above

27. The vital characteristics of first-rate CPR are…. [20].

a. starting chest compressions within 10 seconds of recognition of cardiac arrest

b. pushing hard and fast

c. minimizing interruptions

d. all of above

28. The sign of severe airway obstruction includes all of the following Except … [21].

a. poor air exchange

b. high-pitched noise while inhaling

c. unable to cry

d. may wheeze between coughs

29. While using the AED if the shock is not advised we should …. [22].

a. presses the reanalyze button

b. check pulse

c. immediately resumes CPR

d. check if the pads are attached properly

30. An unconscious patient should be placed in the recovery position after… [23].

a. If the patient has bradycardia and is not breathing

b. If the patient has a pulse and is breathing appropriately

c. If the patient has no pulse and is not breathing

d. If the patient has a bounding pulse and is not breathing

**References used to design questionnaires**

1. Almesned A, Almeman A, Alakhtar AM, AlAboudi AA, Alotaibi AZ, Al-Ghasham YA, Aldamegh MS. Basic life support knowledge of healthcare students and professionals in the Qassim University. Int J Health Sci. 2014;8(2):141.
2. Hazinski MF, Nolan JP, Billi JE, Böttiger BW, Bossaert L, de Caen AR, Deakin CD, Drajer S, Eigel B, Hickey RW, Jacobs I. Part 1: executive summary: 2010 international consensus on cardiopulmonary resuscitation and emergency cardiovascular care science with treatment recommendations. Circulation. 2010;122(16_suppl_2):S250-75.
3. Field RA, Soar J, Davies RP, Akhtar N, Perkins GD. The impact of chest compression rates on quality of chest compressions–a manikin study. Resuscitation. 2012;83(3):360-4.
4. Saquib SA, Al-Harthi HM, Khoshhal AA, Shaher AA, Al-Shammari AB, Khan A, Al-Qahtani TA, Khalid I. Knowledge and attitude about basic life support and emergency medical services amongst healthcare interns in university hospitals: a cross-sectional study. Emerg Med Int. 2019;2019.
5. Koster RW, Baubin MA, Bossaert LL, Caballero A, Cassan P, Castrén M, Granja C, Handley AJ, Monsieurs KG, Perkins GD, Raffay V. European Resuscitation Council Guidelines for Resuscitation 2010 Section 2. Adult basic life support and use of automated external defibrillators. Resuscitation. 2010;81(10):1277-92.
6. Kwiecień-Jaguś K, Mędrzycka-Dąbrowska W, Galdikienė N, Via Clavero G, Kopeć M. A cross-international study to evaluate knowledge and attitudes related to basic life support among undergraduate nursing students—a questionnaire study. Int J Environ Res Public Health. 2020 ;17(11):4116.
7. Hagberg C, Georgi R, Krier C. Complications of managing the airway. Best Pract Res Clin Anaesthesiol. 2005;19(4):641-59.
8. Jo S, Lee JB, Jin Y, Jeong T, Yoon J, Park B, Jung JM. Changes in peak expiratory flow rates using two head-tilt/chin-lift maneuver angles in young healthy conscious volunteers. Plos one. 2019 ;14(10):e0224155.
9. Roppolo LP, Pepe PE, Campbell L, Ohman K, Kulkarni H, Miller R, Idris A, Bean L, Bettes TN, Idris AH. Prospective, randomized trial of the effectiveness and retention of 30-min layperson training for cardiopulmonary resuscitation and automated external defibrillators: The American Airlines Study. Resuscitation. 2007 ;74(2):276-85.
10. Thrush DN. Cardiac arrest after oxymetazoline nasal spray. J Clinic Anesthesia. 1995 ;7(6):512-4.
11. Watkins Jr L, Mirowski M, Mower MM, Reid PR, Griffith LS, Vlay SC, Weisfeldt ML, Gott VL. Automatic defibrillation in man: the initial surgical experience. Jo Thorac Cardiovasc Surg. 1981;82(4):492-500.
12. Atkins DL, Kerber RE. Pediatric defibrillation: current flow is improved by using" adult" electrode paddles. Pediatrics. 1994;94(1):90-3.
13. Marsden AK. Basic life support: revised recommendations of the Resuscitation Council (UK). BMJ: Br Med J. 1989;299(6696):442-
14. Teeter JG, Bleecker ER. Relationship between airway obstruction and respiratory symptoms in adult asthmatics. Chest. 1998 Feb 1;113(2):272-7.
15. Atkins DL, Jorgenson DB. Attenuated pediatric electrode pads for automated external defibrillator use in children. Resuscitation. 2005 ;66(1):31-7.
16. Kanstad BK, Nilsen SA, Fredriksen KC. CPR knowledge and attitude to performing bystander CPR among secondary school students in Norway. Resuscitation. 2011 ;82(8):1053-9.
17. Berg MD, Schexnayder SM, Chameides L, Terry M, Donoghue A, Hickey RW, Berg RA, Sutton RM, Hazinski MF. Part 13: pediatric basic life support: 2010 American Heart Association guidelines for cardiopulmonary resuscitation and emergency cardiovascular care. Circulation. 2010;122(18_suppl_3):S862-75.
18. Root CW, Deutsch BC, Lakha S, Shah A, Lin HM, Hyman JB. Feasibility of a modified strategy for 2-rescuer cardiopulmonary resuscitation. J Emerg Medi. 2019;57(1):51-8.
19. Farsi Z, Yazdani M, Butler S, Nezamzadeh M, Mirlashari J. Comparative effectiveness of simulation versus serious game for training nursing students in cardiopulmonary resuscitation: a randomized control trial. Int J Comput Games Technol. 2021 Mar 24;2021.
20. Kessing LV, Thomsen AF, Mogensen UB, Andersen PK. Treatment with antipsychotics and the risk of diabetes in clinical practice. Br J Psychiatry. 2010;197(4):266-71.
21. Morice RC, Ece T, Ece F, Keus L. Endobronchial argon plasma coagulation for treatment of hemoptysis and neoplastic airway obstruction. Chest. 2001 Mar 1;119(3):781-7.
22. Woollard M, Whitfield R, Smith A, Colquhoun M, Newcombe RG, Vetter N, Chamberlain D. Skill acquisition and retention in automated external defibrillator (AED) use and CPR by lay responders: a prospective study. Resuscitation. 2004;60(1):17-28.
23. Turner S, Turner I, Chapman D, Howard P, Champion P, Hatfield J, James A, Marshall S, Barber S. A comparative study of the 1992 and 1997 recovery positions for use in the UK. Resuscitation. 1998;39(3):153-60.
